# Research Review: What we have learned about early detection and intervention of borderline personality disorder

**DOI:** 10.1111/jcpp.70011

**Published:** 2025-07-14

**Authors:** Michael Kaess, Marialuisa Cavelti

**Affiliations:** ^1^ University Hospital of Child and Adolescent Psychiatry and Psychotherapy University of Bern Bern Switzerland; ^2^ Department of Child and Adolescent Psychiatry, Centre for Psychosocial Medicine University Hospital Heidelberg Heidelberg Germany

**Keywords:** Personality disorder, adolescents, youth, diagnosis, early intervention, psychotherapy

## Abstract

**Background:**

Borderline personality disorder (BPD) typically emerges during adolescence and early adulthood and has severe personal, social and economic consequences. Despite significant research efforts on early intervention over the past two decades, delays in diagnosis and treatment are still common, and exclusion of individuals with BPD from mental health services is prevalent.

**Methods:**

In order to bridge the gap between research and clinical practice, this review qualitatively synthesises empirical evidence on early intervention for BPD, addressing four key questions: (1) Should BPD be diagnosed in adolescents? (2) How is BPD diagnosed in adolescents? (3) Is adolescent BPD treatable, and how effective are treatments? and (4) Can BPD development be prevented?

**Findings:**

Evidence supports diagnosing BPD in adolescents from the age of 12 years, with validated diagnostic measures available. While outpatient, disorder‐specific psychotherapy has shown efficacy in reducing BPD symptoms and self‐harm in youth, the evidence is limited by the small number of randomised controlled trials (RCTs), small sample sizes, heterogeneous inclusion criteria, varying control interventions and high risk of bias. Indicated prevention targeting subthreshold BPD symptoms shows promise, but further research is needed on selective and universal prevention strategies.

**Conclusions:**

Enhancing healthcare professionals' knowledge about early diagnosis and treatment for BPD appears necessary in order to reduce the reluctance to diagnose the disorder in adolescence, which is recommended by many national treatment guidelines today. Additionally, large‐scale, rigorous RCTs are necessary to establish the superiority of disorder‐specific treatments over standard care and explore novel service models that offer easily accessible and scalable evidence‐based care for young people with BPD features.

## Introduction

### Phenomenology and prevalence of BPD

Borderline personality disorder (BPD) is characterised by dysregulation across the interpersonal, cognitive, affective and behavioural domains, with wide heterogeneity in individual clinical presentation (see Table 1; Bohus et al., 2021; Leichsenring et al., 2023). It is estimated to affect approximately 1%–3% of the adult population (Eaton & Greene, 2018; Ellison, Rosenstein, Morgan, & Zimmerman, 2018), with similar prevalence rates in adolescents (Bernstein et al., 1993; Zanarini et al., 2011, 2017). Within clinical settings, however, prevalence rates are notably higher, with youth diagnosed with BPD representing approximately 11% of the outpatient population (Chanen et al., 2004; Chanen, Jovev, Djaja, et al., 2008), up to 50% of inpatients (Grilo et al., 1996) and 76% of adolescents attending the emergency department for suicidal behaviours (Greenfield et al., 2015). A recent Danish study found BPD diagnoses in child and adolescent care for youth under 18 years rose from 23.6% in 2007 to 34.1% in 2017 (Klinkby et al., 2024). These figures underscore the point that individuals with BPD are active help‐seekers and the disorder's impact on healthcare resources. In terms of gender ratio, community‐based samples indicate a relatively balanced prevalence of BPD between males and females (Guilé, Zavaglia, Berthiaume, & Bergeron, 2021; Zanarini et al., 2011). This contrasts with clinical samples, where the female to male ratio is approximately 3:1 (Aouidad et al., 2020; Yen, Gagnon, & Spirito, 2013). This discrepancy indicates potential gender‐related differences in treatment seeking behaviour and diagnostic biases.

**Table 1:** BPD symptomatology (adapted from Gunderson, Herpertz, Skodol, Torgersen, & Zanarini, 2018)

Interpersonal instability	Cognition/self‐disturbance	Emotional dysregulation	Behavioural dysregulation
Unstable relationships characterised by intense involvement and significant withdrawal Alternations between idealisation and devaluation of others Difficulties in acknowledging the emotions and needs of others Hypersensitivity to perceived social threat Intense fear of abandonment, indicated by clinginess, threating or persistent checking	Unstable self‐image Low self‐esteem Pervasive feelings of shame, self‐loathing and guilt Inconsistent, fluctuating goals and values Instances of paranoid ideation or dissociative symptoms	Emotional instability, with individuals responding with heightened sensitivity to daily life stressors Intensely felt and expressed emotions, such as anger, anxiety or sadness Severe and often inappropriate anger outbursts	Compromised impulse control, manifesting in actions that pose risks to the individuals Spending, risky sexual activities, substance misuse, reckless driving, gambling, nonsuicidal self‐injury and recurrent suicidal gestures, threats or attempts

### Aetiological pathways of BPD

From a developmental psychopathology perspective, the aetiology of BPD is best understood by the dynamic interplay between biological and environmental factors that influence self‐ and interpersonal functioning (e.g., emotion regulation, identity development and social cognition) during key developmental stages (Chanen & Kaess, 2012; Chanen, Sharp, Nicol, & Kaess, 2022; Winsper, 2018). BPD in adolescents has a heritability estimate ranging from 30% to 66%, suggesting a moderate genetic predisposition (Belsky et al., 2012; Bornovalova, Hicks, Iacono, & McGue, 2009; Wertz et al., 2020). However, gene variants are likely nonspecific for BPD, hinting at a broader genetic vulnerability to psychiatric disorders (Witt et al., 2017). Temperament traits, such as frequent tantrums or demanding behaviours (Stepp & Lazarus, 2017) or emotion regulation difficulties during childhood (Vogel et al., 2024), may increase the likelihood of early adverse life experiences, including childhood maltreatment (Robin et al., 2021), early bonding impairments (Fleck et al., 2021), harsh, inconsistent parenting or lack of parental warmth (Stepp, Whalen, et al., 2014) or parental invalidation (Franssens, Abrahams, Brenning, Van Leeuwen, & De Clercq, 2021). These experiences can impede the development of effective emotion coregulation and the transmission of social knowledge between the child and caregivers (Winsper, 2018). The interplay between genetic predisposition and early negative interpersonal experiences can further alter brain structures and functions – particularly within prefronto‐limbic pathways (Chanen, Velakoulis, Carison, et al., 2008; Höper et al., 2024; Koenig, Höper, et al., 2021), the peripheral stress response systems (Koenig, Thayer, & Kaess, 2021) or the hypothalamic–pituitary–adrenal axis (Drews, Fertuck, Koenig, Kaess, & Arntz, 2019). During later childhood and adolescence, the individual may encounter additional invalidating social experiences, such as peer bullying, which may eventually lead to the maladaptive traits constituting BPD (Winsper, Hall, Strauss, & Wolke, 2017). Disruptions in early emotional regulation and social cognition – stemming from the interplay between genetic predispositions and environmental experiences – have been identified as key determinants of BPD, informing the two most influential theoretical and psychotherapeutic frameworks for the disorder (Chanen, Sharp, et al., 2022; Winsper, 2018): Linehan's biosocial model (Crowell, Beauchaine, & Linehan, 2009) and the mentalisation‐based model proposed by Fonagy and colleagues (Luyten, Campbell, Allison, & Fonagy, 2020).

### Course of BPD

Precursors for BPD can already be evident in childhood, such as temperamental factors and the cooccurrence of internalising (e.g., anxiety and depression) and externalising symptoms (e.g., disruptive behaviour disorders) (De Fruyt & De Clercq, 2014). BPD usually first manifests by early adolescence, peaks around mid to late adolescence and then largely attenuates over the adult years (Bornovalova et al., 2009; Cohen, Crawford, Johnson, & Kasen, 2005; Johnson, Cohen, Kasen, et al., 2000; Kaess, Brunner, & Chanen, 2014; Stepp, Keenan, Hipwell, & Krueger, 2014; Winsper, 2021). In contrast, a recent meta‐analysis found a peak of BPD symptoms at the age of 29.4 years, rather than through mid–late adolescence (Aleva, Laceulle, Denissen, Hessels, & van Aken, 2022). However, cross‐sectional data only was used, as longitudinal data that was collected was too limited to conduct meaningful testing. Authors also noted that the interpretation of the identified peak is uncertain and that statistical limitations might account for some of the results. Adolescents with BPD are more likely to present with ‘acute’ symptoms (such as impulsive and affective instability), than with the more ‘chronic’ symptoms (such as identity disturbance, unstable relationships and fears of abandonment) that dominate the manifestation in adulthood (Videler, Hutsebaut, Schulkens, Sobczak, & van Alphen, 2019). In support of this, it was found that particularly a combination of risk‐taking and self‐harming behaviour may represent a marker for BPD in adolescence Blaha et al. 2024. Although symptomatic remission is commonly observed (Gunderson, 2011; Zanarini, Frankenburg, Reich, & Fitzmaurice, 2012), research in both adults and adolescents indicates that impairments in psychosocial functioning and quality of life may persist for years (Alvarez‐Jimenez et al., 2014; Alvarez‐Tomás et al., 2017; Skodol et al., 2005; Wertz et al., 2020; Wright, Zalewski, Hallquist, Hipwell, & Stepp, 2016; Zanarini, Frankenburg, Hennen, Reich, & Silk, 2005).

### Rationale for early diagnosis and treatment of BPD

BPD is among the most disabling mental disorders. Typically emerging during adolescence, BPD can hinder young people's ability to meet developmental milestones (Sharp & Wall, 2018), contributing to long‐term negative personal, social and economic consequences. Individuals diagnosed with BPD face an increased risk of physical health issues (El‐Gabalawy, Katz, & Sareen, 2010) and premature mortality, with a suicide rate of approximately 6% (Temes, Frankenburg, Fitzmaurice, & Zanarini, 2019). The burden on families and caregivers is substantial (Courey, Hyndman, Sheasgreen, & McCay, 2021), and the economic costs of BPD are high, arising from both direct treatment expenses and significant indirect costs (van Asselt, Dirksen, Arntz, & Severens, 2007). Thus, given its high prevalence in clinical settings, association with help‐seeking behaviour and significant impact on individuals, families and society, BPD is a prime target for early diagnosis and treatment (i.e., ‘early intervention’) (Chanen & McCutcheon, 2013). As BPD typically emerges during adolescence and young adulthood, this period represents a critical window of opportunity for early intervention. The primary aim of early intervention is to mitigate or prevent the negative long‐term outcomes associated with BPD and promote more adaptive developmental pathways in youth with (subthreshold) BPD features or a first manifestation of the disorder (Chanen, Nicol, Betts, & Thompson, 2020). Delayed diagnosis increases the risk of misdiagnosis, inadequate treatment and iatrogenic harm (Tedesco, Day, Lucas, & Grenyer, 2023) and affects treatment efficacy, particularly regarding functional outcomes (Chanen, 2015; Chanen & McCutcheon, 2013).

### Aims of the current review

Over the past two decades, significant research efforts have focused on early intervention in BPD. With the aim of increasing awareness of the importance of early intervention for BPD and bridging the gap between research and clinical practice, this article provides a qualitative synthesis of the empirical evidence on the following four key questions:

1. Should we diagnose BPD in adolescents?
2. How is BPD diagnosed in adolescents?
3. Is BPD in adolescents treatable, and if so, how effective are available treatments?
4. Can we prevent the development of BPD?

## Early diagnosis of BPD

### Research question 1: Should we diagnose BPD in young people?

There is an ongoing debate whether or not BPD should be diagnosed in young people, with pro and con voices among professionals (Allison, Bastiampillai, Looi, & Mulder, 2022; Cavelti, Sharp, Chanen, & Kaess, 2023; Chanen, 2022; Garralda, 2022; Kingsley, 2022; Schmeck, 2022; Tyrer, 2022) and people with lived experiences (Cannon & Gould, 2022; Courtney & Makinen, 2016; Hartley et al., 2022; Tedesco et al., 2023). In the following, we examine the most common arguments against BPD diagnosis prior to the age of 18 in the light of current empirical evidence (Sharp, 2017).

#### BPD features are not normative developmental adolescent behaviours

It has been argued that BPD features merely reflect typical adolescent behaviour, such as engaging in risky behaviours or experiencing pronounced mood swings. While risk‐taking behaviours and mood fluctuations may increase during adolescence, they tend to normalise as individuals transition into early adulthood (Collado, Felton, MacPherson, & Lejuez, 2014). However, for a clinically significant portion of adolescents, BPD symptoms persist into adulthood (Cohen et al., 2005; Johnson, Cohen, Smailes, et al., 2000), highlighting the importance of identifying and addressing such features before maladaptive patterns of personality pathology become entrenched.

#### Adolescents' personalities are not too unstable to warrant a personality disorder (PD) diagnosis

Concerns that the evolving personalities of adolescents are too unstable for a PD diagnosis are countered by research showing that adolescent personality is neither more nor less stable than adult personality (Roberts & DelVecchio, 2000). Rank‐order stability of maladaptive personality traits is moderate for adolescents and adults, meaning that an individual's ranking among other individuals in terms of PD pathology remains relatively stable over time (Bornovalova, Hicks, Iacono, & McGue, 2013; Chanen et al., 2004). However, less stable in both adolescents and adults are categorical PD diagnoses, with research showing high rates of remission over time (Álvarez‐Tomás, Ruiz, Guilera, & Bados, 2019; Gunderson, 2011), contradicting traditional views of PD as a persistent condition.

#### BPD is not better explained by internalising and externalising disorders

Due to its high comorbidity with mental disorders (see below ‘Differential Diagnosis’), it has been argued that BPD is better explained by internalising and externalising disorders. Research indicates that BPD is neither an internalising disorder nor is it a female expression of antisocial PD, but is most likely to contain characteristics of both internalising and externalising disorders, while still retaining its independence as a separate disorder (Bailey & Finn, 2020; Eaton et al., 2011; James & Taylor, 2008).

#### BPD is not a sequelae of trauma

A variation of the argument above includes the claim that BPD is better described as a sequelae of trauma (Hartley et al., 2022) or a form of attachment disorder (Chanen, 2021). While patients with BPD indeed experience childhood adversity more often than clinical and healthy controls (Porter et al., 2020), it is overly simplistic to say BPD is solely caused by childhood trauma, which is neither a necessary nor a sufficient precondition (Cavelti et al., 2023; Chanen, 2021).

#### Psychiatric classification systems do allow the diagnosis of BPD in adolescence

It has been questioned whether psychiatric classifications allow the diagnosis of BPD in adolescence (Chanen, 2022). While current psychiatric classifications permit the BPD diagnosis in adolescents, the DSM‐5 recommends diagnosis only in ‘those relatively unusual instances in which the maladaptive personality traits appear to be pervasive, persistent, and unlikely to be limited to a particular developmental stage or another mental disorder’ (p. 648). However, the DSM‐5 Alternative Model for Personality Disorders (AMPD) and the ICD‐11 have completely removed age‐based restrictions for diagnosing PDs.

#### Avoidance of diagnosis does not necessarily reduce stigma

There is a debate about whether it is best to avoid labelling young people with BPD to spare them from long‐lasting stigma (Ring & Lawn, 2019). Indeed, BPD tends to attract more stigma than other mental health conditions, especially among mental health professionals (McKenzie, Gregory, & Hogg, 2022). However, it is unlikely that changing the label will really reduce stigma (Corrigan, Bink, Fokuo, & Schmidt, 2015). Many patients actually prefer open and honest communication about their diagnosis (Lester et al., 2020), and how diagnoses are communicated and the culture of services are crucial determinants in whether a PD diagnosis is perceived as helpful or not (Sulzer, Muenchow, Potvin, Harris, & Gigot, 2016).

Taken together, research supports BPD as a valid and reliable diagnosis in adolescents with distinct treatment implications (Chanen, 2015; Chanen, Jovev, McCutcheon, Jackson, & McGorry, 2008; Miller, Muehlenkamp, & Jacobson, 2008; Sharp & Fonagy, 2015). Therefore, to overcome the myths around ‘the diagnosis that must not be named’ (Cannon & Gould, 2022; Chanen & McCutcheon, 2008; Larrivée, 2013), current national treatment guidelines advocate for diagnosis BPD in individuals aged 12 and older (Deutsche Gesellschaft für Psychiatrie und Psychotherapie, Psychosomatik und Nervenheilunde e.V. (DGPPN), 2022; National Collaborating Centre for Mental Health, 2009; National Health and Medical Research Council, 2012; Simonsen et al., 2019).

### Research question 2: How is BPD diagnosed in adolescence?

#### Diagnostic criteria in current psychiatric classificatory systems

The DSM‐III (American Psychiatric Association, 1987) marked BPD's official inclusion in psychiatric classifications (Gunderson, 2009). Subsequent revisions in the DSM‐IV introduced a ninth diagnostic criterion, focusing on cognitive disturbances like stress‐induced paranoia or severe dissociative episodes (American Psychiatric Association, 1994). The criteria remained consistent through the DSM‐IV‐TR (American Psychiatric Association, 2000) and DSM‐5 (American Psychiatric Association, 2013) updates. A diagnosis of BPD is justified when an individual meets at least five of the nine criteria outlined in Table 2. Although the criteria for diagnosing BPD are the same for adults and adolescents, for those under 18, the symptoms must persist for at least 1 year, whereas for adults, the minimum duration is 2 years.

**Table 2:** Diagnostic criteria according to DSM‐5 section II (American Psychiatric Association, 2013)

1.	Frantic efforts to avoid real or imagined abandonment. (Note: Do not include suicidal or self‐mutilating behaviour covered in Criterion 5.)
2.	A pattern of unstable and intense interpersonal relationships characterised by alternating between extremes of idealisation and devaluation
3.	Identity disturbance: markedly and persistently unstable self‐image or sense of self
4.	Impulsivity in at least two areas that are potentially self‐damaging (e.g., spending, sex, substance abuse, reckless driving and binge eating). (Note: Do not include suicidal or self‐mutilating behaviour covered in Criterion 5)
5.	Recurrent suicidal behaviour, gestures or threats, or self‐mutilating behaviour
6.	Affective instability due to a marked reactivity of mood (e.g., intense episodic dysphoria, irritability or anxiety usually lasting a few hours and only rarely more than a few days)
7.	Chronic feelings of emptiness
8.	Inappropriate, intense anger or difficulty controlling anger (e.g., frequent displays of temper, constant anger and recurrent physical fights)
9.	Transient, stress‐related paranoid ideation or severe dissociative symptoms

The traditional categorical approach to PDs has faced criticism for its high within‐type heterogeneity, significant diagnostic overlap, arbitrary diagnostic thresholds and the assignment of individual PD symptoms to specific disorders that do not correspond to their empirical covariation, often leading to multiple PD diagnoses, a ‘not otherwise specified’ PD diagnosis or no PD diagnosis at all (Mulder & Tyrer, 2019; Widiger & Trull, 2007). This critique has prompted a shift towards a dimensional approach, as seen in the DSM‐5 AMPD (American Psychiatric Association, 2013) and the ICD‐11 (World Health Organization, 2021). The AMPD introduces a hybrid model that identifies BPD by (a) impairments in self (i.e., identity and self‐direction) and interpersonal functioning (i.e., empathy and intimacy) representing the core and severity of PD in general, and (b) maladaptive personality traits (i.e., negative affectivity, detachment, disinhibition, antagonism and psychoticism) representing stylistic differences in the expression of PD (see Table 3 for the proposed BPD criteria according to the AMPD). The ICD‐11 also classifies PDs according to severity of impairments in self‐ and interpersonal functioning, followed by the option of using trait domains (i.e., negative affectivity, detachment, disinhibition, dissociality and anankastia) to describe the individual expression of personality dysfunction. This means that the ICD‐11 eliminates specific PD types. However, it allows specifying a ‘borderline pattern’ that resembles the criteria described in section II of the DSM‐5. Thus, the DSM‐5 AMPD and the ICD‐11 approaches are comparable with respect to both severity and trait domains (except for Psychoticism) that align with well‐established trait models (Bach & Mulder, 2022). Important advantages of the novel dimensional approaches are that they facilitate the detection of subthreshold PD features and change over time, supporting early intervention efforts. While a growing body of research has evaluated the new dimensional models of PD, the DSM‐5 AMPD has received more attention than the ICD‐11 classification. However, due to the similarities between these frameworks, it has been argued that findings from AMPD research are largely generalisable to the ICD‐11 PD model (Mulder, 2021). Research on the AMPD has provided evidence supporting the validity and reliability of Criterion A (Morey, McCredie, Bender, & Skodol, 2022) and Criterion B (Clark & Watson, 2022), as well as the overall clinical and prognostic utility of the AMPD (Bach & Tracy, 2022). Despite these advancements, the conceptualisation and relative importance of Criterion A and B remain subjects of ongoing debate (Hopwood, 2022; Livesley, 2022; Sleep & Lynam, 2022). Additionally, most research in this area, with a few exceptions (Balzen, Sharp, Unzurruzaga, Eguren, & Pérez, 2024; Boone, Babinski, Kujawa, Pegg, & Sharp, 2025; Hertel et al., 2024; Thomson et al., 2024; Weekers, Hutsebaut, Rovers, & Kamphuis, 2024; Weekers, Verhoeff, Kamphuis, & Hutsebaut, 2021; Wyssen et al., 2024), has been conducted on adult samples. Key priorities for future research include: refining and improving the conceptualisation and measurement of both Criterion A and Criterion B; demonstrating the AMPD's superiority over the DSM‐5 Section II categories; providing evidence to justify retaining the six traditional PD categorical constructs; harmonising the AMPD with the ICD‐11 framework; validating the model's applicability for younger populations; developing informant‐based measures suitable for youth samples; and employing AMPD measures as primary outcomes in clinical trials (Sharp & Miller, 2022; Vanwoerden & Stepp, 2022; Widiger & Hines, 2022; Zimmerman, 2022).

**Table 3:** BPD criteria according to the DSM‐5 AMPD

A	Moderate or greater impairment in personality functioning, manifested by characteristic difficulties in two or more of the following four areas:
1.	Identity: Markedly impoverished, poorly developed or unstable self‐image, often associated with excessive self‐criticism; chronic feelings of emptiness; dissociative states under stress
2.	Self‐direction: Instability in goals, aspirations, values or career plans
3.	Empathy: Compromised ability to recognise the feelings and needs of others associated with interpersonal hypersensitivity (i.e., prone to feel slighted or insulted); perceptions of others selectively biased towards negative attributes or vulnerabilities
4.	Intense, unstable and conflicted close relationships, marked by mistrust, neediness and anxious preoccupation with real or imagined abandonment; close relationships often viewed in extremes of idealisation and devaluation and alternating between overinvolvement and withdrawal
B	Four or more of the following seven pathological personality traits, at least one of which must be (5) impulsivity, (6) risk taking or (7) hostility:
1.	Emotional lability (an aspect of negative affectivity): Unstable emotional experiences and frequent mood changes; emotions that are easily aroused, intense and/or out of proportion to events and circumstances
2.	Anxiousness (an aspect of negative affectivity): Intense feelings of nervousness, tenseness or panic, often in reaction to interpersonal stresses; worry about the negative effects of past unpleasant experiences and future negative possibilities; feeling fearful, apprehensive or threatened by uncertainty; fears of falling apart or losing control
3.	Separation insecurity (an aspect of negative affectivity): Fears of rejection by – and/or separation from – significant others, associated with fears of excessive dependency and complete loss of autonomy
4.	Depressivity (an aspect of negative affectivity): Frequent feelings of being down, miserable and/or hopeless; difficulty recovering from such moods; pessimism about the future; pervasive shame; feelings of inferior self‐worth; thoughts of suicide and suicidal behaviour
5.	Impulsivity (an aspect of disinhibition): Acting on the spur of the moment in response to immediate stimuli; acting on a momentary basis without a plan or consideration of outcomes; difficulty establishing or following plans; a sense of urgency and self‐harming behaviour under emotional distress
6.	Risk taking (an aspect of disinhibition): Engagement in dangerous, risky and potentially self‐damaging activities, unnecessarily and without regard to consequences; lack of concern for one's limitations and denial of the reality of personal danger
7.	Hostility (an aspect of antagonism): Persistent or frequent angry feelings; anger or irritability in response to minor slights and insults

#### Assessing BPD in adolescents

The gold standard for diagnosing BPD is the use of semi‐structured clinical interviews. Several interviews that have been specifically developed for adolescents or at least validated in individuals less than 18 years old, exist. In addition, self‐report questionnaires can be applied for screening purposes to identify those for which a specific diagnostic interview is warranted or to assess the severity of BPD (for reviews, see Chanen et al., 2020; Fonagy et al., 2015; Sharp & Fonagy, 2015). Table 4 presents the most frequently used interviews and self‐report questionnaires for assessing BPD in young people according to the DSM‐5 section II diagnostic criteria. In addition, an increasing number of instruments are available to assess PD according to the new dimensional conceptualisations in the DSM‐5 AMPD and ICD‐11 (for reviews, see Bach et al., 2022; Zimmermann, Kerber, Rek, Hopwood, & Krueger, 2019). However, only a limited number of these instruments have been validated for use with younger populations, limiting their utility for early detection efforts. Notable examples of tools suitable to assess personality pathology in young people include the Semi‐Structured Interview for Personality Functioning DSM‐5 (STiP‐5.1; Hutsebaut, Kamphuis, Feenstra, Weekers, & De Saeger, 2017; Thomson et al., 2023); the Levels of Personality Functioning Questionnaire for adolescents aged 12 to 18 (LoPF‐Q 12‐18; Goth, Birkhölzer, & Schmeck, 2018); the Levels of Personality Functioning Questionnaire Parent Rating (LoPF‐Q 6‐18 PR; Mazreku et al., 2023); and the Personality Inventory for DSM‐5 (PID‐5; De Clercq et al., 2014). These examples underscore the need for further research to expand the availability and validation of instruments tailored to younger populations.

**Table 4:** Measures to assess BPD in youth according to the DSM‐5 section II diagnostic criteria

Measure	Method
Child Interview for DSM‐IV Borderline Personality Disorder (CI‐BPD) (Michonski, Sharp, Steinberg, & Zanarini, 2013; Sharp, Ha, Michonski, Venta, & Carbone, 2012; Zanarini, 2003)	Structured interview
Revised Diagnostic Interview for Borderline (DIB‐R) (Zanarini et al., 2017)	Structured interview
BPD Severity Index IV Adolescent Version (BPDSI‐IV‐Adolescent) (Schuppert, Bloo, Minderaa, Emmelkamp, & Nauta, 2012)	Structured interview
Structured Clinical Interview for DSM‐IV Axis II Personality Disorders (SCID‐II) (Chanen & McCutcheon, 2008; First, Spitzer, Gibbon, Williams, & Benjamin, 1997; Kaess, Fischer‐Waldschmidt, Resch, & Koenig, 2017)	Structured interview
Borderline Personality Features Scale for Children (BPFSC) (Crick, Murray‐Close, & Woods, 2005; Sharp, Mosko, Chang, & Ha, 2011; Sharp, Steinberg, Temple, & Newlin, 2014)	Self‐report/parent‐report
BPD items of the SCID‐II Personality Questionnaire (SCID‐II‐PQ) (Chanen, Jovev, Djaja, et al., 2008)	Self‐report
Borderline Personality Questionnaire (BPQ) (Chanen, Jovev, Djaja, et al., 2008; Henze et al., 2013; Poreh et al., 2006)	Self‐report
McLean Screening Instrument for Borderline Personality Disorder (MSI‐BPD) (Chanen, Jovev, Djaja, et al., 2008; Noblin, Venta, & Sharp, 2014; Van Alebeek, Van Der Heijden, Hessels, Thong, & Van Aken, 2017; Zanarini et al., 2003)	Self‐report
Borderline Symptom List (BSL) (Bohus et al., 2009; Mehlum et al., 2014; Shen et al., 2023)	Self‐report

#### Differential diagnosis

In adolescents with BPD, comorbidity with mental disorders is rather the norm than the exception, necessitating a comprehensive diagnostic evaluation across a broad range of psychopathology (Choate, Fatimah, & Bornovalova, 2021). Common co‐occurring mental disorders in adolescents with BPD are affective disorders (both unipolar and bipolar), anxiety disorders, eating disorders, dissociative disorders, attention deficit hyperactivity disorders (ADHD), oppositional defiant/conduct disorders (ODD/CD) and substance use disorders (Chanen, Berk, & Thompson, 2016; Guilé et al., 2021; Kaess, von Ceumern‐Lindenstjerna, et al., 2013; Speranza et al., 2011). Additionally, psychotic symptoms and even co‐occurring psychotic disorders can occur in youth with BPD (Cavelti et al., 2019; Cavelti, Thompson, Chanen, & Kaess, 2021; Chanen et al., 2024; Thompson, Cavelti, & Chanen, 2019). Notably, a significant proportion of adolescents with BPD meets criteria for complex comorbidity, defined as the co‐occurrence of internalising and externalising disorders (Chanen, Jovev, & Jackson, 2007; Ha, Balderas, Zanarini, Oldham, & Sharp, 2014; Zanarini et al., 2021).

Recently, complex posttraumatic stress disorder (cPTSD) has been introduced in the ICD‐11, which exhibits conceptual similarities with BPD regarding difficulties in affect regulation, self‐concept and interpersonal relationships (Ford & Courtois, 2021). A key diagnostic criterion distinguishing cPTSD from BPD is the requirement of exposure to a traumatic stressor for the former, which is not a necessity for the latter. Furthermore, empirical studies have identified distinctive features between the two disorders, including self‐harming and impulsive behaviour (more common in BPD than in cPTSD), self‐concept (unstable sense of self in BPD versus persistent negative view of self in cPTSD) and relationship difficulties (volatile, intense interaction pattern in BPD versus persistent avoidance and social withdrawal in cPTSD) (Maercker et al., 2022).

The most dominant explanation for the comorbidity of BPD and common mental disorders is the *p*‐factor, capturing communal liabilities across disorders or broader transdiagnostic domains (Forbes, Tackett, Markon, & Krueger, 2016; Oltmanns, Smith, Oltmanns, & Widiger, 2018; Shields, Giljen, España, & Tackett, 2021). Alternatively, the co‐occurrence of BPD and mental disorders may emerge from the developmental interplay between lower‐level symptoms (i.e., internalising and externalising features), as proposed by dynamic mutualism theory (Choate et al., 2021).

## Early treatment and prevention of BPD

### Research question 3: Is BPD in adolescents treatable, and if so, how effective are available treatments?

Until the end of the last century, both researchers and clinicians mostly endorsed the view that PD (including BPD) is an untreatable life‐span condition (i.e., therapeutic nihilism) (Klein, Fairweather, & Lawn, 2022). In line with this, early detection and treatment were neither recommended nor implemented in mental healthcare systems worldwide. To date, however, there is increasing evidence that BPD is a changeable and thus treatable condition (Cavelti et al., 2023; Chanen, Sharp, et al., 2022; Hutsebaut, Clarke, & Chanen, 2023; Kaess et al., 2014). For instance, recent longitudinal data from early intervention cohorts of adolescents with BPD pathology show marked improvements in both BPD pathology as well as secondary outcomes such as self‐harm or psychosocial functioning (Cavelti, Seiffert, et al., 2024). In addition, there is evidence for the effectiveness of early intervention from the age of 12 years, but with an age‐dependent manifestation: preventing the normative increase of BPD pathology expected in younger adolescents, and significantly decreasing BPD pathology in older adolescents (Kaess et al., 2024). As a conclusion of the current evidence, several national guidelines (e.g., the German AWMF guidelines, the Australian NHMRC guidelines and the UK Nice guidelines) now recommend early treatment of BPD in adolescents (from the age of 12 or 14 years). While there is – as outlined below – emerging evidence for the potential of early intervention for BPD, it is important to note that the current evidence is often based on a low number of studies from few early intervention centres worldwide, posing a serious limitation to the generalisability of current knowledge.

As with any other mental health condition, the treatment of BPD typically involves a multimodal approach that encompasses various service components and types of interventions.

#### Service components

Most evidence for early intervention to date is derived from specialised outpatient clinics for early intervention of BPD, for example, the HYPE clinic in Melbourne, Australia (Chanen et al., 2009), the AtR!Sk clinics in Bern, Switzerland and Heidelberg, Germany (Kaess, Ghinea, Fischer‐Waldschmidt, & Resch, 2017) or the Viersprong Institute for Studies on Personality Disorders in the Netherlands (this may not be an exhaustive list but highlights a selection of notable examples). They usually provide comprehensive diagnostic assessment and evidence‐based individual psychotherapy along with general psychiatric management for those who present with at least three BPD criteria (Chanen & Thompson, 2018). There are various forms of individual psychotherapy that have been evaluated in RCTs (see below ‘Psychotherapy’). However, more and more experts stress the importance of developing and disseminating common quality standards of basic mental healthcare for young people with BPD in order to improve accessibility of effective early intervention (Hutsebaut, Willemsen, Bachrach, & Van, 2020). This approach is supported by the results of a recent randomised controlled trial (RCT) that compared specialised outpatient treatment for BPD (with or without individual psychotherapy) to standardised service delivery by a general youth mental health service and found no evidence for superiority, neither of the specialised early intervention programme nor the specific individual psychotherapy in terms of psychosocial improvements (Chanen, Betts, et al., 2022). Results indicated that effective early intervention was not reliant on availability of specialist psychotherapy but did require a non‐stigmatising and nonharmful service culture, youth‐oriented clinical case management and psychiatric care. While this finding certainly needs replication and noninferiority of good general psychiatric management still needs to be confirmed in larger RCTs, the current evidence at least seems to support the notion that structured standard care is far better than rejecting adolescents with BPD from urgently needed mental healthcare. Thus, we should encourage clinicians and mental health services worldwide to develop and implement structured child and adolescent psychiatric care that adheres to a few major principles of BPD treatment (see e.g., good clinical management; Ilagan & Choi‐Kain, 2021) for the purpose of early intervention of BPD.

An efficient setting to provide structured psychotherapy is the group setting. While groups are standard components of some disorder‐specific psychotherapies for youth with BPD (e.g., Dialectical Behaviour Therapy for Adolescents; DBT‐A), it remains unclear whether they are as beneficial as a stand‐alone treatment. A recent RCT aiming to prove the effectiveness of a group therapy using Mentalisation‐Based Treatment for Adolescents (MBT‐A) did not find differences in BPD symptom reduction compared to a treatment as usual (TAU) group (Beck et al., 2020).

A further important question of service delivery concerns inpatient treatment. Inpatient treatment – in particular involuntary inpatient care – is still very common among youth with BPD, mostly due to their impulsive high‐risk and chronic self‐harming or suicidal behaviour. Experts in the field agree that inpatient treatment bears risks for young individuals with BPD, including the potential to further exacerbate symptoms (e.g., self‐harming behaviour) and increase functional deficits, promoting long‐term dependency on the healthcare system (Kaess, Herpertz, Plener, & Schmahl, 2019). However, RCTs on the comparison between inpatient and outpatient care are lacking, mainly for feasibility and ethical reasons. Nonetheless, a recent study on an early intervention cohort compared those adolescents who only received outpatient care with those who received both inpatient and outpatient treatment. Even after adjustment for a large variety of potentially confounding variables, the ‘outpatient only’ group showed better outcomes within the two‐year follow‐up period, which was most pronounced for psychosocial functioning (Cavelti, Seiffert, et al., 2024).

An intermediate setting that has gained some attention in the field is the use of day clinics among adolescents with BPD. While first evaluations revealed promising symptom reduction (Gilbey et al., 2023), RCTs in adolescents are lacking, and a recent trial in adults was not able to prove superiority compared to specialist treatment as usual in an outpatient setting (Laurenssen et al., 2018).

Overall, current evidence suggests that specialised outpatient care is beneficial for early intervention of adolescents with BPD, and inpatient care should be offered with maximal caution. There is some data pointing to a beneficial effect of good clinical management in those adolescents as well, which may represent a promising avenue for future research and service delivery. The role of intermediate care or stand‐alone group settings still needs further clarification.

#### Psychotherapy

Psychotherapy is regarded the first‐line treatment for early intervention in youth with BPD to date. However, before going into the evidence in detail, a recent meta‐analysis investigating the effectiveness of psychotherapy for adolescents with BPD concluded that due to heterogeneous samples and interventions, the high risk of bias, high attrition rates and underpowered studies in this area, ‘it is difficult to derive any conclusions on the efficacy of psychological therapies for BPD in adolescence’ (Jørgensen et al., 2021). Thus, we need to acknowledge that the evidence that will now be presented still needs replication and confirmation in future RCTs with larger samples and rigorous methodology. Overall, two meta‐analyses have summarised the effectiveness of psychotherapy specifically for adolescents with BPD diagnosis or features. Wong, Bahji, and Khalid‐Khan (2020) investigated the overall treatment effects of BPD‐specific treatments (4 studies included) and found significant effects regarding the reduction of overall BPD symptom severity (Hedges'*g* = −0.89, 95% CI −1.75, −0.02) and self‐harm (OR = 0.34, 95% CI 0.16, 0.74). It is important to note that the included trials included adolescents fulfilling a minimum of two BPD criteria only, so that they did not solely investigate individuals with full‐syndrome BPD. The meta‐analysis by Jørgensen et al. (2021) included 10 RCTs (for a brief description see Table 5) with each of the included samples comprising more than 70% individuals with full diagnosis of BPD. In this analysis, the authors reported results specific to the type of psychotherapy (e.g., MBT‐A and DBT‐A). In the meta‐analysis on BPD severity as primary outcome (including four trials of MBT and emotion regulation training (ERT)), no evidence of an effect was found. Results from individual studies indicated that a significant reduction of BPD severity was observed in one DBT‐A trial and one MBT‐A trial. No other RCT demonstrated superiority compared to the respective control condition (mostly TAU). However, the evidence for both DBT‐A (Mehlum et al., 2014) and MBT‐A (Rossouw & Fonagy, 2012) was based on data from only one study each, and the treatment effect of DBT‐A on BPD severity was not sustained over time (no difference between DBT‐A and control condition at 1‐year and 3‐year follow‐up) (Mehlum et al., 2016, 2019). The potential effectiveness of DBT‐A in reducing a variety of BPD symptoms was also previously confirmed by an uncontrolled treatment study that revealed a reduction of all nine BPD criteria during outpatient DBT‐A (Buerger et al., 2019). For self‐harm, the meta‐analysis by Jørgensen et al. (2021) found a statistically pooled significant effect (OR = 0.45, 95% CI 0.26, 0.76) exclusively for DBT‐A, and only at end of treatment (two trials). The long‐term effects of DBT‐A on self‐harm in individual studies yielded mixed results: Mehlum et al. (2016, 2019) demonstrated that DBT‐A had sustained benefits on self‐harm at 1‐year and 3‐year follow‐ups, while McCauley et al. (2018) reported no significant differences between DBT‐A and the control condition at 6‐month follow‐up. The effectiveness of DBT‐A in reducing self‐harm was recently corroborated by a meta‐analysis conducted by Kothgassner et al. (2021). The analysis revealed small to moderate effects on self‐harming behaviour (*g* = −0.44; 95% CI −0.81 to −0.07) and suicidal ideation (*g* = −0.31, 95% CI −0.52 to −0.09) from pre to postintervention.

**Table 5:** Description of included trials in the meta‐analysis by Jørgensen et al. (2021) on psychotherapies for adolescent BPD

Trial	*n*	Setting	Age	No. of BPD criteria	Intervention group	Control group
Chanen, Jackson, McCutcheon, et al. (2008)	*n* = 86 (76% female)	Outpatient	16.4 (*SD* = 0.9)	41% BPD, 59% ≥ 2	CAT	SGCC
Schuppert et al. (2009)	*n* = 43 (88.4% female)	Outpatient	16.14 (*SD* = 1.23)	ns ≥ 2	ERT + TAU	TAU
Schuppert, Timmerman, et al. (2012)	*n* = 109 (96% female)	Outpatient	15.98 (*SD* = 1.22)	73% BPD, 27% ≥ 2	ERT + TAU	TAU
Gleeson et al. (2012)	*n* = 16 (81.2% female)	Outpatient	18.4 (*SD* = 2.9)	75% BPD, 25% ≥ 4	CAT + SFET	SFET
Rossouw and Fonagy (2012)	*n* = 80 (85% female)	Outpatient	14.7 (*SD* = ns)	73% BPD, 27% no. ns	MBT‐A	TAU
Mehlum et al. (2014)	*n* = 77 (88.3% female)	Outpatient	15.6 (*SD* = 1.5)	20.5% BPD, 79.5% ≥ 2 + self‐harm	DBT‐A	EUC
Salzer, Cropp, Jaeger, Masuhr, and Streeck‐Fischer (2014)	*n* = 39 (female ns)	Inpatient (experimental), Outpatient (control)	ns	100% BPD	PiM	WL/TAU
Santisteban et al. (2015)	*n* = 40 (37.5% female)	Outpatient	15.8 (*SD* = 0.8)	100% BPD	I‐BAFT	IDC
McCauley et al. (2018)	*n* = 173 (94.8% female)	Outpatient	14.89 (*SD* = 1.47)	53.2% BPD, 46.8% ≥ 3	DBT‐A	IGST
Beck et al. (2020)	*n* = 112 (99.1% female)	Outpatient	15.8 (*SD* = 1.1)	96% BPD, 4% ≥ 4	MBT‐G	TAU

Abbreviations: CAT, Cognitive analytic therapy; DBT‐A, Dialectical behaviour therapy for adolescents; ERT, Emotion regulation training; EUC, Enhanced usual care; I‐BAFT, Integrative borderline personality disorder‐oriented adolescent family therapy; IDC, Individual drug counselling; IGST, Individual and group supportive therapy; MBT‐A, Mentalisation‐based treatment for adolescents; MBT‐G, Mentalisation‐based treatment in groups; ns, not specified; PiM, Psychoanalytic‐interactional method; SFET, Specialist first episode psychosis treatment; SGCC, Standardised good clinical care; TAU, Treatment as usual; WL, Waiting list.

Besides the potential of disorder‐specific psychotherapy approaches, brief interventions that target symptoms that are particularly prominent in emerging BPD (e.g., nonsuicidal self‐injury; NSSI) may also be beneficial. An RCT investigating the effectiveness of a brief psychotherapeutic intervention specifically targeting adolescent self‐harm found that individuals with BPD showed equal reduction of NSSI compared to those without BPD (Kaess et al., 2020). The beneficial effect of this intervention on the long‐term trajectory of BPD symptoms could also be demonstrated during a two‐ to four‐year follow‐up of the trial (Rockstroh et al., 2023), suggesting that a brief targeted intervention on self‐harm may be an effective first step within a potential stepped‐care approach to early intervention of BPD. Following these findings, a recent study evaluated the stepped‐care model for early intervention of BPD applied at the Heidelberg and Bern AtR!Sk outpatient clinics that offer a brief psychotherapeutic intervention addressing NSSI to all patients, followed by a more intensive DBT‐A for those whose symptoms persist. The results support the decision criteria of three BPD criteria for the offer of a more intense therapy after the short‐term psychotherapy but did not find evidence for the efficacy of DBT‐A as step‐up treatment (Cavelti, Blaha, et al., 2024).

Overall, the current evidence suggests that psychotherapeutic interventions are capable of reducing BPD symptoms during early intervention of BPD in youth (mostly with large effect sizes in symptom reduction) but the evidence for the superiority of disorder‐specific treatments is limited (e.g., the meta‐analysis by Jørgensen et al. (2021) found evidence exclusively for DBT‐A, and this conclusion was based on data from a single study). While future research will need to invest in more large‐scale and methodologically rigorous RCTs, the finding that most RCTs failed to outperform TAU (e.g., Chanen, Betts, et al., 2022; Jørgensen et al., 2021) may support the current ideas of good clinical management (including brief structured psychotherapy or even none at all) in early intervention of BPD. In addition, new research studies will be needed to investigate the effectiveness of psychotherapy on adolescent BPD according to the new dimensional conceptualisations of PD in the AMPD and the ICD‐11.

#### Medication

Medication is still a common feature of multimodal treatment in BPD among adolescents. A recent analysis of three RCTs primarily conducted to investigate DBT‐A in Norway, the United States and Spain revealed a heterogeneous spectrum of pharmaceutical treatment in 12% (Norway) up to 86% (Spain) of the samples (Mehlum et al., 2024). The most common medications used were antidepressants and mood stabilisers. Most importantly, the use of medication was rarely correlated to co‐occurring diagnoses, such as major depression, and the different prescribing strategies could not be explained by differences in sample characteristics. These findings suggest that current treatment practices are neither fully empirically supported nor do they reflect current treatment guidelines. Another study that investigated intervention components for the treatment of adolescents with BPD in several European university settings found that 76.5% were treated with at least one psychopharmacological substance (more than 50% were using several medication types) while only 47% of patients received psychotherapy (Cailhol et al., 2013).

These findings point to a common problem in the treatment of BPD: the use of (poly)pharmacotherapy, despite the lack of evidence for its efficacy. Recent meta‐analyses in adults confirmed that there is no evidence for an effect of any psychotropic medication on the reduction of BPD pathology (Gartlehner et al., 2021; Stoffers‐Winterling et al., 2022). For adolescents, rigorous RCTs on any medication are lacking. Interestingly, a recent nationwide register study from Sweden investigating adult patients who received treatment for BPD revealed that previous treatment with ADHD medication was associated with a reduced risk of psychiatric rehospitalisation or hospitalisation owing to any cause or death (Lieslehto et al., 2023). There were no beneficial findings for any other medication such as benzodiazepines, antidepressants, antipsychotics or mood stabilisers.

In sum, empirical evidence for pharmacotherapy in early intervention of BPD is lacking, and medication should be prescribed with maximal caution, in particular if the target symptoms relate to BPD. Nonetheless, there may still be a beneficial effect of pharmacotherapy for the treatment of common co‐occurring mental disorders (e.g., depression and ADHD). Further research should explore the role of pharmacotherapy in the treatment of common comorbidities of BPD, as well as the potential of ADHD medication for the treatment of BPD features (e.g., emotion dysregulation).

### Research question 3: Can we prevent BPD?

In light of increasing mental health problems among youth (see e.g., for the US 2022 National Healthcare Quality and Disparities Report, 2022), as well as rising healthcare costs in many countries worldwide (World Health Organization, 2023), prevention of mental disorders will be critical to reduce the overall burden of disease in the future. There are different types of prevention that can be implemented at different levels of healthcare systems: universal prevention (targeting all individuals of a given population) typically includes public health campaigns or school‐based prevention programmes; selective prevention (targeting individuals with specific risk factors) can be implemented in schools (e.g., through social workers or school psychologists) but can also be offered by easily accessible healthcare providers (e.g., community care settings or psychosocial counseling services); and indicated prevention (targeting individuals who already seek help with emerging symptoms of the disorder) is commonly part of early intervention efforts in clinical care settings.

#### Indicated prevention

The goal of indicated prevention is to target and effectively reduce early emerging symptoms of a disorder before it develops into a full syndrome. Indicated prevention is the first step of early intervention (Mrazek & Haggerty, 1994) and is, to date, an integral part of most early intervention efforts worldwide, including those for BPD. The rationale behind these efforts is the idea that mental disorders (e.g., in some way similar to oncological conditions) show clinical progression into more severe stages of illness over time and that clinical staging should be applied to adapt interventions to the respective severity of illness (Scott et al., 2024).

Recently, staging models have been proposed for BPD (see Table 6; Hutsebaut, Videler, Verheul, & Van Alphen, 2019), which recommend the implementation of indicated prevention at stage 1. In most studies, this stage is operationalised as three or more emerging BPD features and commonly labelled as ‘subclinical’, ‘subthreshold’ or ‘at‐risk’. It is important to note that indicated prevention typically targets individuals who are help‐seeking and already present with a substantial burden of illness (i.e., high psychopathological distress and reduced quality of life; Kaess, Fischer‐Waldschmidt, Resch, & Koenig, 2017).

**Table 6:** Staging model for BPD, adapted from Hutsebaut et al. (2019)

Stage	Borderline features	Comorbidity	Social and occupational functioning
Stage 0	No classic symptoms of BPD, but latent impairments in self and interpersonal functioning, expressed in problems in mood regulation, attention deficits, frustration and distress tolerance	Either no formal disorders or some areas of mental problems, including ADHD, conduct problems	No extensive problems, but areas of problems, including school functioning or peer contacts
Stage I	Emerging symptoms of BPD, usually in the areas of affect dysregulation and impulse control	Usually ‘comorbid’ disorders, including mood, anxiety and conduct disorders	Emerging significant problems in school, peer contacts or relationship between parents and child
Stage II	First episode of full BPD	Usually comorbid disorders, often in associated areas of emotion dysregulation (mood disorders, PTSD, substance abuse)	Significant and lasting problems in school, peer contacts and family
Stage III	Relapse in full BPD or chronic patterns of full BPD	Usually chronic and multiple comorbid disorders	Usually recurring significant problems in social and occupational functioning
Stage IV	Full BPD without remission of main problem areas	Usually severe and chronic associated psychopathology	No social or occupational functioning

The evidence for indicated prevention for BPD is largely similar to the evidence of early intervention detailed above (see ‘Research question 3: Is BPD in adolescents treatable, and if so, how effective are available treatments?’). This is because most studies on early intervention for BPD have included subthreshold forms of the disorder (see Table 5). This means that current approaches of disorder‐specific psychotherapy appear to also have promise in reducing symptoms of emerging BPD, thereby stopping the progression into full‐syndrome BPD.

One important open question related to the suggested staging model is whether there is a dose response relationship of treatment that corresponds with the respective clinical stage. In a traditional staging model (e.g., for cancer), earlier stages of the disorder can be treated successfully with less invasive interventions and lower treatment doses, whereas later stages of the disease require more intensive and invasive treatments. Translated to early intervention for BPD, this would mean that subclinical presentations of BPD (stage 1) could be treated by less intensive interventions (e.g., brief psychotherapy) compared to full‐syndrome BPD (stage 2) and that patients in stage 2 would not sufficiently benefit from low‐dose interventions. Recent data on brief psychotherapy (Rockstroh et al., 2023) suggest that brief interventions can be equally effective for individuals with full‐syndrome BPD as those with subthreshold BPD, which may in parts question the proposed staging approach. However, a comprehensive evaluation of the staging model's assumptions for early intervention in BPD remains lacking – particularly one that incorporates the new dimensional conceptualisations of PD and considers broader outcomes beyond symptomatic remission, such as psychosocial functioning. Integrating such an evaluation into a transdiagnostic staging model that addresses a broader spectrum of co‐occurring psychopathology could provide valuable insights (Scott et al., 2024). Overall, and in line with the evidence for early treatment of full‐syndrome BPD, current evidence and international guidelines recommend BPD‐specific treatment also for indicated prevention. There is some data that suggest that early stages of BPD can be successfully treated by low‐dose interventions, but this may also be true for later stages, and more research will be needed to explore the idea of stage‐specific care for adolescents with BPD. In addition, the new dimensional diagnostic classifications for PD (and BPD; see ‘Diagnostic criteria in current psychiatric classificatory systems’) will certainly influence the entry criteria to both early intervention and indicated prevention in the decade to come.

#### Selective prevention

Past research has revealed a variety of important precursors and risk factors for BPD development (see ‘Aetiological pathways of BPD’). However, to date, there is no evidence for the effectiveness of selective prevention interventions in reducing the onset of BPD. Nonetheless, we would like to outline a few potential targets for selective prevention programmes that could be explored in the future for their preventive impact on BPD. First, precursor symptoms of BPD such as children's temperament (Fleck et al., 2021; Kaess, Resch, et al., 2013) or childhood symptoms of BPD (Fleck et al., 2023) could be identified and targeted by individual as well as family interventions. Second, early signs of psychopathology in childhood (e.g., ADHD, anxiety and conduct disorder/oppositional defiant disorder) that are predictive of BPD development in adolescence (e.g., Stepp, Olino, Klein, Seeley, & Lewinsohn, 2013) could be targeted by routine clinical care and evaluated regarding its potential for preventing BPD development. Third, parental mental disorders (including BPD) and parents' childhood adversity (e.g., Infurna, Fuchs, et al., 2016) could be assessed to select high‐risk groups for parent–child relationship problems (see below). Fourth, early parent–child relationship problems (e.g., Williams, Fleck, Fuchs, Koenig, & Kaess, 2023), including bonding difficulties (e.g., Fleck et al., 2021), seem promising as potential targets for family early interventions that may prevent BPD development among many other mental health problems. Finally, childhood abuse and neglect should be prevented or rigorously identified given that it is still among the most important predictors for BPD development (e.g., Infurna, Brunner, et al., 2016). Further research is essential to determine whether the outlined targets are truly disorder‐specific risk factors or primarily transdiagnostic in nature. If they are found to be transdiagnostic, they may be more appropriately addressed through universal prevention strategies.

#### Universal prevention

So far, there is no data on universal prevention efforts for BPD. This is not entirely surprising given that it is probably neither feasible nor appropriate to specifically prevent single disorders with rather low prevalence and largely overlapping developmental pathways via universal prevention. Nonetheless, it would be interesting to investigate whether BPD symptomatology may be prevented by general universal interventions that do not specifically target BPD, but rather address shared risk factors (e.g., childhood maltreatment) or improve mental well‐being by building resilience and enhancing protective factors (e.g., emotion regulation skills and social support).

## Discussion

This literature review on the current evidence base of early detection and treatment for BPD revealed a solid evidence base for early diagnosis, while the findings on the effectiveness of early treatment are mixed and limited by a low number of RCTs, small sample sizes, varying inclusion criteria and control interventions, and a high risk of bias (Jørgensen et al., 2021).

Given the solid evidence base for early detection of BPD, the primary need is to bridge the research‐practice gap. This primarily involves training healthcare professionals, from primary to specialist care, to improve their understanding of BPD, dispel myths about the disorder and address discomfort with the label, as well as prejudicial and discriminatory attitudes and behaviours. Such training will ensure timely diagnosis and access to evidence‐based treatment for the affected young people and their families. Additionally, community campaigns should aim to increase mental health literacy and reduce stigma, including BPD, which is among the most stigmatised mental disorders (Hazell, Berry, Bogen‐Johnston, & Banerjee, 2022). This appears necessary, as fear of stigma remains a major barrier to professional help‐seeking among youth (Cavelti, Ruppen, et al., 2024). Research priorities should focus on the development and evaluation of brief, user‐friendly and potentially developmentally adapted assessments with a particular focus on the new dimensional conceptualisations of BPD, facilitating their systematic use in both clinical research and routine practice.

In terms of early treatment of BPD, there is a need for large‐scale, methodologically rigorous RCTs evaluating disorder‐specific psychological interventions (Jørgensen et al., 2021), but also pharmacological treatment for both BPD‐specific symptoms and frequent comorbid conditions. Beyond measuring remission of clinical symptoms, these trials should comprehensively quantify the educational, vocational and social long‐term outcomes and provide detailed health economic data (Chanen et al., 2017). However, given the increasing demands on mental health services, financial cutbacks and staff shortages, the focus should be on treatments that are easily scalable to enhance accessibility to evidence‐based care. This includes developing and scientifically evaluating novel service models that stratify treatment according to the current illness stage and needs of patients, and incorporate digital technologies (Uhlhaas et al., 2023). When developing and evaluating these service models, it is crucial to integrate BPD with other severe mental health disorders, recognising the ‘equifinal’ and ‘multifinal’ pathways in the development of psychopathology (Shah, Jones, van Os, McGorry, & Gülöksüz, 2022). Moreover, young people and their families should be included as partners in designing these new service models to ensure they meet their specific needs and reduce the high dropout rates typically seen in treatments for BPD (Arntz et al., 2023; Iliakis, Ilagan, & Choi‐Kain, 2021). While currently indicated prevention targeting individuals with subthreshold features of BPD and aiming to stop the development of the full‐blown disorder presents the most promising, feasible and cost‐effective strategy (Chanen et al., 2017), it may also be worthwhile to explore selective and universal preventive strategies in the future.

## Funding

MC is supported by a grant from the Swiss National Science Foundation (SNSF; PZ00P1_193279).

**Key points**

- BPD is a severe mental disorder that typically emerges during adolescence and young adulthood, with serious long‐term consequences for individuals, families and societies.
- Contrary to persistent beliefs that the disorder cannot or should not be diagnosed in adolescents, current empirical evidence shows that BPD can be reliably and validly diagnosed from the age of 12 onwards. Early diagnosis is crucial to prevent delayed access to evidence‐based treatment.
- Although research on the effectiveness of early treatment for BPD is increasing, the evidence base remains limited due to a small number of RCTs, small sample sizes, heterogeneous samples and interventions and publication bias. However, current evidence supports outpatient, specialist treatment as the first‐line treatment for youth with BPD features or a first manifestation of the full syndrome.
- Major efforts are needed to enhance healthcare professionals' knowledge about the early diagnosis of BPD in adolescents and to develop novel services that offer easily accessible and scalable treatments that meet the special needs of young people with BPD pathology.
